# Supplementary material for: A Targeted Metagenomics Approach to Study the Diversity of Norovirus GII in Shellfish Implicated in Outbreaks
Source: Viruses. 2020 Sep 3;12(9):978. doi: 10.3390/v12090978 (PMC7552006; doi:10.3390/v12090978)
Supplement: Supplementary file 1 [file viruses-12-00978-s001.pdf]

Table S1. Genotype or P-type and accession number of NoV references used for mapping.

Gene	Classification	Accession number
RdRp	GII.P1	U07611
	GII.P2	X81879
	GII.P3	U22498
	GII.P4_1995	AB089860
	GII.P4_2002	AY502023
	GII.P4_2002CN	EU31092
	GII.P4_2003	AB294779
	GII.P4_2004	EF126961
	GII.P4_2006a	EF126963
	GII.P4_2006b	EF126966
	GII.P4_2007EU	AB49129
	GII.P4_2007JP	GQ84536
	GII.P4_2009	JN595867
	GII.P4_Brist	X76716
	GII.P4_Camb	AF145896
	GII.P5	AF397156
	GII.P6	AB039778
	GII.P7	AB258331
	GII.P8	AB039780
	GII.P11	AB126320
	GII.P12	AB220922
	GII.P12	AF504671
	GII.P13	EU921354
	GII.P15	GQ856474
	GII.P16	AY682551
	GII.P17	LC037415
	GII.P18	AY823304
	GII.P20	EU424333
	GII.P21	AY682549
	GII.P22	AB083780
	GII.P23	MG495080
	GII.P24	KY225989
	GII.P25	MG495083
	GII.P26	KU306738
	GII.P27	MG495077
	GII.P28	KJ196291
	GII.P29	AB190457
	GII.P30	AY134748
	GII.P31	JX459907
	GII.P32	AY682550
	GII.P33	GQ845370
	GII.P34	AB089882

	GII.P35	AY682552
	GII.P36	AF315813
	GII.P37	EU921353
	GII.P38	GQ856469
	GII.P39	FJ537134
	GII.P40	AB212306
	GII.P41	DQ379714
	GII.PNA1	MG495079
	GII.PNA2	MG706448
	GII.PNA3	FJ537135
	GII.PNA4	AB074893
	GII.PNA5	MG495082
	GII.PNA7	LC342059
	GII.PNA8	KR904229
	GII.PNA9	MK733205
VP1	GII.1	U07611
	GII.2	X81879
	GII.3	U22498
	GII.4_1995	AF080558
	GII.4_2002	AY502023
	GII.4_2002CN	EU310927
	GII.4_2003	AB303929
	GII.4_2004	DQ078814
	GII.4_2005	DQ369797
	GII.4_2006a	EF126963
	GII.4_2006b	EF684915
	GII.4_2007Ca	GQ845368
	GII.4_2007EU	HQ009513
	GII.4_2007JP	AB434770
	GII.4_2009	JN400623
	GII.4_2012	JX459908
	GII.4_2016	LC175468
	GII.4_2019	CUHK_NS_22
	GII.4_Bristol	X76716
	GII.4_Cambridge	AF145896
	GII.5	AF397156
	GII.6	AB039778
	GII.7	AF414409
	GII.8	AB039780
	GII.9	AY038599
	GII.10	AY237415
	GII.11	AB126320
	GII.12	EU921353
	GII.13	EU921354
	GII.14	GQ856465

	GII.16	AY772730
	GII.17	LC037415
	GII.18	AY823304
	GII.19	AY823306
	GII.20	EU424333
	GII.21	GQ856468
	GII.22	AB083780
	GII.24	KY225989
	GII.25	GQ856474
	GII.26	KU306738
	GII.NA1	MG495079
	GII.NA2	MG706448
	GIII.1	EU360814
	GIV	EU224456
	GIX.1	GQ856474
	GVIII.1	KJ196291
